# Treatment of intraoperatively detected peritoneal carcinomatosis of colorectal origin with cytoreductive surgery and intraperitoneal chemotherapy

**DOI:** 10.1186/s12957-018-1369-7

**Published:** 2018-03-27

**Authors:** Aras Emre Canda, Cigdem Arslan, Cem Terzi, Selman Sokmen, Tugba Yavuzsen, Sevda Ozkardesler, Mehtat Unlu, Funda Obuz, Mehmet Fuzun

**Affiliations:** 1 Department of Surgery, Dokuz Eylul University School of Medicine, Balcova, 35340 Izmir, Turkey; 2 Department of Medical Oncology, Dokuz Eylul University School of Medicine, Balcova, 35340 Izmir, Turkey; 3 Department of Anaesthesiology and Reanimation, Dokuz Eylul University School of Medicine, Balcova, 35340 Izmir, Turkey; 4 Department of Pathology, Dokuz Eylul University School of Medicine, Balcova, 35340 Izmir, Turkey; 5 Department of Radiodiagnostics, Dokuz Eylul University School of Medicine, Balcova, 35340 Izmir, Turkey

**Keywords:** Peritoneal carcinomatosis, Intraperitoneal chemotherapy, HIPEC, EPIC, Colorectal, Incidental, Intraoperative detected

## Abstract

**Background:**

Diagnosis of peritoneal carcinomatosis (PC) may be missed by preoperative imaging. We are presenting our experience with incidentally detected PC of colorectal origin treated with cytoreductive surgery (CRS) and intraperitoneal chemotherapy (IPC) at the same operation.

**Methods:**

Between January 2010 and September 2016, 19 patients underwent CRS and IPC due to incidentally detected PC of colorectal origin. Data were analyzed from a prospectively collected database.

**Results:**

The median age was 59 (29–78). In three patients, PC was diagnosed during emergency surgery. The primary tumor was located in the rectum (three patients; one with recurrent disease), left colon (9 patients), and right colon (7 patients). All patients underwent CRS and IPC, and one patient operated laparoscopically. Median peritoneal cancer index (PCI) was 5 (range, 3–14), and complete cytoreduction (CC-0) was achieved in 14 patients. After CRS, 8 patients received early postoperative intraperitoneal chemotherapy (EPIC), 7 patients received hyperthermic intraperitoneal chemotherapy (HIPEC), and 4 patients received both HIPEC and EPIC. The median hospital stay was 9 (6–29) days. Postoperative complications occurred in 6 patients. There was no postoperative mortality. Median follow-up was 40.2 (12–94) months. Five-year overall survival was 63.2%. Estimated mean survival time is longer in patients who underwent complete cytoreduction compared to patients having CC-1 or CC-2 cytoreduction (87.7 vs. 20.3 months; *p* < 0.001).

**Conclusion:**

Cytoreductive surgery and IPC can be performed safely in patients with intraoperatively detected incidental PC of colorectal origin.

## Background

Peritoneal carcinomatosis (PC) from colorectal cancer (CRC) has a poor prognosis and often considered as a terminal condition. Overall survival with current systemic chemotherapy regimens with new chemotherapeutic and molecular targeting agents varies between 13 and 34 months [1–3]. Currently, long-term survival can only be achieved by cytoreductive surgery (CRS) and intraperitoneal chemotherapy (IPC). The incidence of synchronous PC in patients with colorectal cancer is 7% [4]. Despite the advancements in imaging technics, the diagnostic accuracy of radiology in the identification of PC is still unsatisfying especially in patients having low-volume disease. There are no recommendations in the guidelines or consensus reports for the management of the patients with unexpected peritoneal metastasis during surgery for CRC. In this study, we present our results of CRS and IPC in patients who underwent surgery for CRC with no preoperative suspicion of peritoneal metastasis.

## Methods

### Patients’ characteristics

Between January 2010 and September 2016, we incidentally detected PC of colorectal origin in 24 patients during intraoperative exploration and performed CRS and IPC. We excluded patients with unresectable disease. Although all of the patients had preoperative computed tomography (CT) scans, we could not identify PC preoperatively. Peritoneal metastasis was confirmed in all patients, during working hours by frozen section and out of working hours by histopathology. In our department, we can perform CRS and IPC, and we routinely inform the patients for the need of multivisceral resections or CRS whom undergoing oncological surgery. Our main criteria for considering a patient unsuitable for CRS and IPC are the presence of diffuse small bowel or periportal involvement, unable to perform CC-0 and CC-1 cytoreduction, and extensive distant metastasis. The patients’ performance status evaluated individually.

### Cytoreductive surgery

The objective during CRS is to remove of all macroscopically visible tumor nodules from the visceral and parietal peritoneum by resection of the effected organ/tissues or with peritonectomy procedures as previously described by Sugarbaker [5]. Electrosurgery was used for implants on visceral or intestinal surfaces where resection or excisions of the nodules were not possible. The extent of the peritoneal involvement was measured by peritoneal cancer index (PCI) [6]. After the completion of the resections and peritonectomy procedures, “Completeness of Cytoreduction” (CC) was classified as CC-0, no residual disease; CC-1, minimal residual disease of 0–2.5 mm; CC-2, residual disease of 2.5 mm–2.5 cm; and CC-3, residual disease > 2.5 cm [7].

### Intraperitoneal chemotherapy

The rationale for performing IPC is to extend macroscopic disease elimination achieved by CRS to microscopic disease elimination. After the completion of cytoreduction, we delivered hyperthermic intraperitoneal chemotherapy (HIPEC) under general anesthesia with closed abdominal technique. Two inflow (one in the deep pelvis, one in the subhepatic or mostly affected area) and two outflow drains (one in the superficial pelvis site cavity) and two thermal probes were positioned in the abdominal cavity. The abdominal wall or skin was closed, although, after completion of the HIPEC, the surgical team would be able to re-explore or create gastrointestinal continuity at this site, if necessary. The drains and thermal probes were connected to the extracorporeal circuit of the HIPEC machine (Performer LRT, Rand, Italy). Three to five liters of perfusate were used depending on the abdominal cavity volume. Our oncologist coordinated chemotherapeutic dose for every individual patient. We used oxaliplatin at a dose of 430 mg/m^2^ at 42–43 °C intracavitary temperature for 30 min for peritoneal metastases from the colorectal origin. For early postoperative intraperitoneal chemotherapy (EPIC), we placed four outflow drains at the same position as in HIPEC and a Jackson–Pratt drain subhepatic space as an inflow catheter. In the surgical ward, a peritoneal infusion in 1 l of 0.09 NaCl was given at day 0 from the Jackson–Pratt drain in order to prevent intraperitoneal adhesions. Then, 5-FU (650 mg/m^2^) and sodium bicarbonate in 1 l of 0.09 NaCl were given intraperitoneally in the next 5 days. These drugs remained in place for 23 h before drainage for 1 h before the next infusion.

### Evaluation of complications and toxicity

According to our protocol for CRS and IPC, we record complications, systemic toxicities, and mortality occurring during the postoperative hospital stay or within 30 days of operation. We retrospectively analyzed those data.

### Oncological follow-up

Follow-up included a physical examination and CEA measurements every 3 months for the first year, twice a year afterward. A CT scan of the abdomen and thorax every 6 months for the first 2 years and yearly after that. We perform colonoscopy at the end of year 1. Magnetic resonance imaging or positron emission CT is not routine imaging tools and performed when necessary. The exact status of each patient was retrospectively analyzed from a specific database of Surgery and Oncology Departments.

### Statistical analysis

Continuous variables were expressed as means and minimum and maximum values (range) and categorical variables as frequency and percentages. Patients’ data were compiled into a computer statistical software including demographic, surgical, pathologic, and survival figures. Survival rates were calculated using Kaplan–Meier method and were compared with the log-rank test (*p* < 0.05 was considered statistically significant).

## Results

**Fig. 1:**
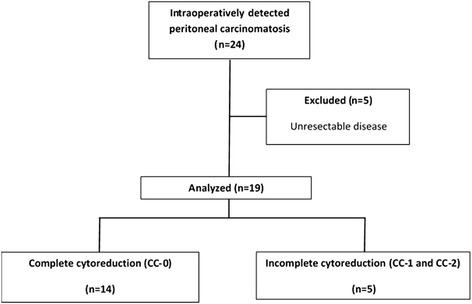
Flow diagram of the study

Nineteen patients were included in the analysis. Flow diagram of the study is given in Fig. 1. The median age of the patients was 59 (range; 29–78) years, and five of them were female. Five patients had comorbidities including chronic obstructive pulmonary disease (one patient), diabetes mellitus (two patients), and hypertension (three patients). Median American Society of Anesthesiologists (ASA) score was 1 (range, 1–3). The primary tumor was located in the rectum (three patients), left colon (9 patients), and right colon (7 patients). Two patients with locally advanced rectal cancer received preoperative chemoradiotherapy. One patient underwent surgery for recurrent rectal cancer; others had a primary disease. In three patients, PC was diagnosed during emergency surgery. All patients underwent CRS and IPC, and one patient operated laparoscopically. We performed Hartmann procedure in 2 patients and diverting loop ileostomy in one patient which was closed to 6 following the initial procedure. Median PCI was 5 (range, 3–14). We achieved macroscopically complete cytoreduction (CC-0) in 14 patients. After completion of CRS, we performed IPC, EPIC in 8 patients, HIPEC in 7 patients, and both HIPEC and EPIC in four patients. None of the patients stayed in intensive care unit; the median hospital stay was 9 days (range, 6–29) (Table 1).

**Table 1:** Demographic and surgical characteristics of the patients

Variables	
Sex	
Male	14
Female	5
Age (year)	59 (29–78)
ASA score	1 (1–3)
Tumor origin	
Right colon	7
Left colon	9
Rectum	3
Median PCI score	5 (3–14)
Completeness of cytoreduction	
CC-0	14
CC-1	4
CC-2	1
Type of intraperitoneal chemotherapy	
EPIC	8
HIPEC	7
EPIC + HIPEC	1
Median hospital stay (days)	9 (6–29)

*ASA* American Society of Anesthesiologists, *PCI* peritoneal carcinomatosis index, *EPIC* early postoperative intraperitoneal chemotherapy, *HIPEC* hyperthermic intraperitoneal chemotherapy

Postoperative complications occurred in 6 patients. These included surgical site infection in four patients, urinary tract infection in two patients, chylous drainage in one patient, and small intestine perforation in one patient (she underwent a reoperation). No WHO grade 3 or 4 bone marrow or renal toxicities were observed. We did not find any difference related to different types of IPC regarding postoperative complications. No patients died during the perioperative period.

Based on the histological examination, primary tumor stage was T3 in three patients and T4 in 16 patients and 10 patients had lymph node metastases. Postoperatively, all patients received further systemic chemotherapy.

**Fig. 2:**
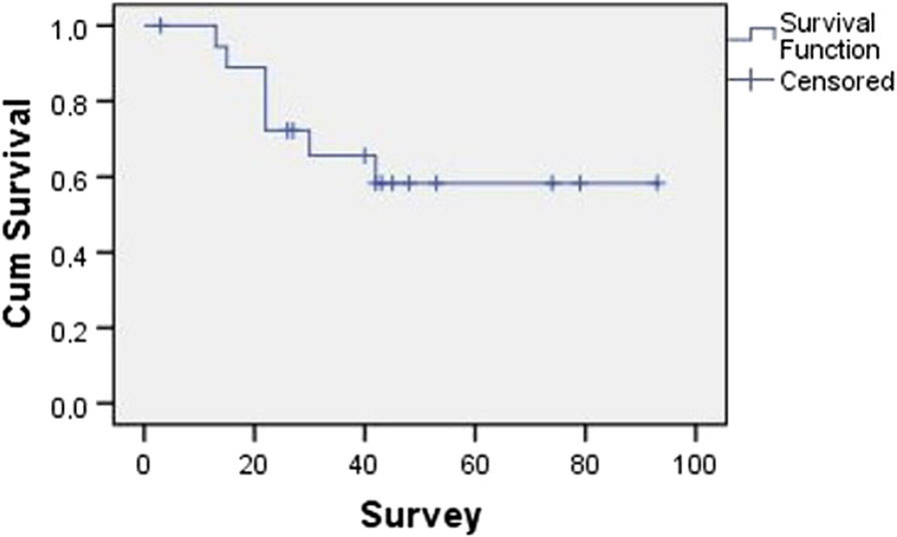
Kaplan–Meier survival curve for median survival

**Fig. 3:**
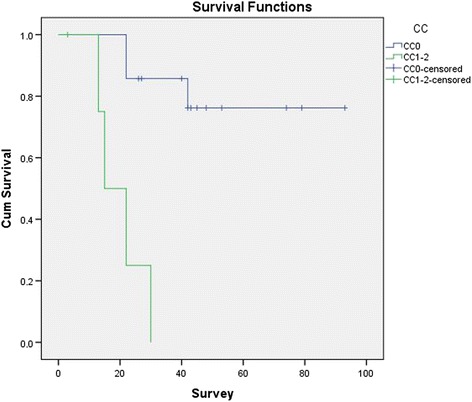
Kaplan–Meier survival curves following complete and incomplete cytoreduction

Median follow-up time was 40.2 (range, 3–94) months. Two patients developed isolated local recurrence, four patients developed isolated distant metastasis, and two patients developed combined local recurrence and distant metastasis. Median time to local recurrence and distance metastasis was 5 (3–14) and 11 (8–24) months. Two patients with CC-1 and CC-2 resections died due to progressive disease; three patients, due to locally recurrent; and two patients, due to metastatic disease. Two patients were alive with persistent disease, and 10 patients were alive without any evidence of disease. Estimated mean (±SE) survival time was 64.5 (±8.4) months with a 5-year survival rate of 63.2% (Fig. 2). Estimated mean survival time is longer in patients who had no lymph node metastasis compared to patients having lymph node metastasis (77.1 vs. 37.8 months); however, the difference did not reach statistical significance (*p* = 0.428). Estimated mean survival time is significantly longer in patients who had complete cytoreduction compared to patients having CC-1 or CC-2 cytoreduction (78 vs. 20 months; *p* < 0.001) (Fig. 3).

## Discussion

The peritoneum is the second most common site after the liver of colorectal cancer metastases [8]. The natural history of the disease has a poor median survival of approximately 6 months which is increased up 34 months with new systemic chemotherapy regimens [1–3, 9, 10]. However, long-term survival is still hard to be achieved by systemic chemotherapy alone. Elias et al. reported 60% 2-year survival with cytoreductive surgery with or without EPIC in patients with CRC and PC [11]. Long-term follow-up results of a randomized controlled trial showed 45% disease-free survival rates in CRS and HIPEC arm compared to less than 10% in incomplete cytoreduction or systemic chemotherapy arm [12]. A recent meta-analysis confirmed the improvement of survival with CRS and HIPEC in selected patients with peritoneal carcinomatosis from colorectal cancer [13]. Peritoneal Surface Oncology Group International (PSOGI) reached a consensus that CRS and HIPEC should be considered as the standard therapy for the selected patients with mild-to-moderate peritoneal metastasis [14]. The addition of EPIC to HIPEC may provide an increase in survival but increases the morbidity [15].

Currently, standard preoperative radiologic tool for staging colorectal cancer is CT [16]. The sensitivity of CT for detecting PC is 60–90% and influenced by the extent of the disease, size, and site of the nodules [17, 18]. Although multi-detector CT enables more accurate images, the extent of the PC is underestimated in approximately one third of the patients [18–20]. The accuracy of CT decreases by the size of the implants, particularly in right-upper quadrant, right-lower quadrant, left-lower quadrant, distal jejunum, and distal ileum [18]. Tumor nodules < 5 mm and small-bowel mesentery location have detection sensitivities as low as 10% with CT [19, 20]. Despite the advancements in imaging technics, we still face with unexpected PC intraoperatively.

There are no clear recommendations or publications for the management of the patient with intraoperatively detected and unexpected peritoneal metastasis of CRC. Closing the abdomen with only a biopsy and reference to a tertiary center having access to HIPEC or preoperative systemic chemotherapy are alternatives. Initial surgery should be as sparing as possible, in order not to damage the peritoneal surface and to evoke intraperitoneal release of growth factors [21]. Unnecessary dissection and resections may result with adhesions which may be a challenge for the surgeon who will going to perform CRS. On the other hand, considering the low tumor burden which could not be detected by CT, most of these patients would be suitable candidates for CRS and IPC. In the presence of experienced surgical team and sufficient technical settings including 7/24 available oncology consultant and chemotherapeutical agents, intraoperatively detected PC can be treated by CRS and IPC at once at the same surgery. In our center, we can offer CRS and IPC for those patients. Several factors influence the choice of IPC. When we detect PC incidentally and if we operate the patient in working hours, we are able to perform a frozen section to confirm the peritoneal metastases and consult the patient intraoperatively with the oncologist, and we can deliver HIPEC. If we detect PC out of the working hours, we are not able to perform a frozen section so we get biopsies from the implants and deliver EPIC after the histopathological confirmation at the following days. Also, reimbursement of HIPEC is a problem in our country and conditions vary, so we perform HIPEC when the patient’s insurance or the patient individually pays. We prefer the addition of EPIC to HIPEC in patients with incomplete cytoreduction.

Postoperative morbidity and mortality have been reported 12–56 and 0–12% in the literature [22]. Overall morbidity and mortality were reported 39.0–48.5 and 6.5–7.6% in previously published studies of our group [23, 24]. In the present study, morbidity was lower than our entire CRS and IPC series, presumably due to lower PCI scores requiring less aggressive surgery and shorter operative times. There was no postoperative mortality.

We diagnosed synchronous PC during emergency surgery for the primary tumor in three patients. Due to low sample size, we did not perform a comparison in terms of operative outcomes, postoperative complications, toxicities, adjuvant therapies, and survival. Van Oudheusden et al. reported their results in patients who underwent CRS and HIPEC after emergency surgery in the presence of PC, and they observed similar operative outcomes, postoperative complications, and survival compared with the patients in whom PC was diagnosed in an elective setting [21]. When performed by a specialized team, CRS and IPC are a safe procedure in selected patients with PC from colorectal origin.

The PCI score and completeness of cytoreduction have been shown to be associated with better survival in several studies [25, 26]. The expected extent of the disease in patients with incidentally found PC is low, insomuch preoperatively undetectable. Therefore, those patients have the best potential to have a curative treatment of PC. In our study, the median PCI was 5, and unsurprisingly, estimated 5-year overall was favorable when compared with the literature [11, 13]. We found significantly longer mean survival in patients who underwent complete cytoreduction compared to the patients having CC-1 or CC-2 cytoreduction.

## Conclusion

There is a demand for management of intraoperatively detected PC. Cytoreductive surgery and IPC can be performed safely in patients with intraoperatively detected incidental PC of colorectal origin. A multidisciplinary team work, on-demand availability of frozen section, intraoperative oncology consultation, and HIPEC machine are essential for intraoperative management of PC. Controlled trials are needed to identify the best timing of definitive treatment.

## Abbreviations

ASA: American Society of Anesthesiologists
CC: Completeness of Cytoreduction
CRC: Colorectal cancer
CRS: Cytoreductive surgery
CT: Computed tomography
EPIC: Early postoperative intraperitoneal chemotherapy
HIPEC: Hyperthermic intraperitoneal chemotherapy
IPC: Intraperitoneal chemotherapy
PC: Peritoneal carcinomatosis
PCI: Peritoneal cancer index
PSOGI: Peritoneal Surface Oncology Group International
